# Responses of an *In Vitro* Coculture Alveolar Model for the Prediction of Respiratory Sensitizers (ALIsens^®^) Following Exposure to Skin Sensitizers and Non-Sensitizers

**DOI:** 10.3390/toxics13010029

**Published:** 2024-12-31

**Authors:** Sabina Burla, Aline Chary, Tommaso Serchi, Sébastien Cambier, Kristie Sullivan, Elizabeth Baker, Nikaeta Sadekar, Arno C. Gutleb

**Affiliations:** 1 Luxembourg Institute of Science and Technology (LIST), 4422 Belvaux, Luxembourg; 2 Faculty of Food Science and Technology, University of Agricultural Sciences and Veterinary Medicine of Cluj-Napoca, 400372 Cluj-Napoca, Romania; 3 Invitrolize Sarl, 4422 Belvaux, Luxembourg; 4 Physicians Committee for Responsible Medicine (PCRM), Washington, DC 20016, USA; 5 Institute for In Vitro Sciences, Inc. (IIVS), Gaithersburg, MD 20878, USA; 6 Research Institute for Fragrance Materials (RIFM), Woodcliff Lake, NJ 07430, USA

**Keywords:** chemical allergy, new approach methodologies, respiratory sensitization, air-liquid-interface exposure, dendritic cell activation, cell surface markers

## Abstract

In recent years, a global increase in allergy incidence following chemical exposure has been observed. While the process of skin sensitization is well characterized through the adverse outcome pathway (AOP) framework, the immunological mechanisms underlying respiratory sensitization remain less well understood. Respiratory sensitizers are classified as substances of very high concern (SVHC) under the European Union (EU) regulation for the registration, evaluation, authorization and restriction of chemicals (REACH), emphasizing the importance of evaluating respiratory tract sensitization as a critical hazard. However, the existing new approach methodologies (NAMs) for the identification of skin sensitizers lack the capacity to differentiate between skin and respiratory sensitizers. Thus, it is imperative to develop physiologically relevant test systems specifically tailored to assess respiratory sensitizers. This study aimed to evaluate the efficacy of ALIsens^®^, a three-dimensional (3D) *in vitro* alveolar model designed for the identification of respiratory sensitizers and to determine its ability to correctly identify sensitizers. In this study, we used a range of skin sensitizers and non-sensitizers to define the optimal exposure dose, identify biomarkers, and establish tentative thresholds for correct sensitizer classification. The results demonstrate that ALIsens^®^ is a promising *in vitro* complex model that could successfully discriminate respiratory sensitizers from skin sensitizers and non-sensitizers. Furthermore, the thymic stromal lymphopoietin receptor (TSLPr) cell surface marker was confirmed as a reliable biomarker for predicting respiratory sensitization hazards.

## 1. Introduction

Allergic reactions arising from chemical exposure represent a significant concern for human health and can manifest in various ways. Exposure to respiratory sensitizers, in particular, can lead to long-term health conditions, including allergic rhinitis [1] and asthma [2]. Respiratory sensitization resulting from chemical exposure occurs less frequently than skin sensitization, largely attributable to a limited number of identified chemical compounds [3], compared to over 3000 molecules identified as skin sensitizers [4], and exposure context from occupational settings to everyday environments. Despite its lower prevalence, respiratory sensitization is critically important due to its significant impact and severity, being associated with considerable morbidity on the affected individuals and related increased socioeconomic costs [5,6].

Regulatory agencies enforce specific guidelines and regulations for the classification and labeling of substances identified as respiratory and skin sensitizers. Early identification of respiratory sensitizers and distinguishing respiratory from skin sensitizers provides the necessary data to evaluate the hazards associated with exposure and for appropriate risk management measures to be implemented for the protection of human health [7]. This information is essential for making informed decisions regarding the safe handling, use and management of these substances. As many respiratory sensitizers are encountered in occupational settings, testing is essential for workers’ protection. Currently, regulatory agencies do not request the testing of chemicals for respiratory sensitization potential during product registration or authorization due to the lack of validated testing methods.

The mechanisms underlying respiratory sensitization are not fully understood [8,9], but the proposed adverse outcome pathway (AOP) for respiratory sensitization [10] shows points of convergence with the AOP for skin sensitization [11], suggesting common mechanisms involved in the development of the two types of sensitization [12]. These relationships between respiratory and skin sensitization are important for the hazard and risk assessment process of chemical sensitizers. It has been observed that all identified respiratory sensitizers yield positive results in the *in vivo* assays designed for skin sensitization and exhibit dermal sensitization, while the opposite is not universally true [13]. Only a minority of skin sensitizers are also respiratory sensitizers. This suggests that if a compound tests negative in validated tests for the identification of skin sensitizers, the likelihood of it being a respiratory sensitizer may be low, but finally, no decision on its respiratory sensitization hazard can be taken [3,14]. However, if the assay for skin sensitization evaluation tests positive, further investigation is required to ascertain the respiratory sensitization hazard [15]. Distinguishing respiratory from skin sensitizers is essential for their correct classification to implement effective risk management, apply targeted safety measures, and reduce health risks.

Identification of low molecular weight (LMW) chemicals that may induce respiratory sensitization is of paramount importance as respiratory sensitizers have been categorized as substances of very high concern (SVHC) due to their severe impacts on human health [16]. When a chemical compound is classified as a SVHC under the Registration, Evaluation, Authorisation and Restriction of Chemicals (REACH) regulation [17], several regulatory consequences follow. The first step is the inclusion of the substance in the candidate list of SVHC [18], which triggers obligations for both manufacturers and importers, such as providing information on the presence of the SVHC in products to downstream users and consumers. The identification as an SVHC often leads to initiatives for substituting it with safer alternatives or phasing out the use of these substances where feasible. In case the European Chemicals Agency (ECHA) moves a substance from the Candidate List to the Authorization List (Annex XIV of REACH Regulation), its use becomes strictly controlled [17]. These measures have a significant impact on the manufacturers, and identification of respiratory sensitizers in the early development phases is very important for a large array of industries for several reasons: to reduce risks associated with SVHCs, to ensure better protection for human health and the environment by promoting safe handling practices to minimize exposure and mitigate risks, ensuring safer work environments and consumer products.

At present, the classification of chemicals as respiratory sensitizers requires evaluating their sensitization potential through the analysis of scientific evidence derived from occupational data, clinical and experimental studies, and read-across approaches [9,19,20,21]. A significant challenge in the process of risk assessment of chemicals inducing respiratory sensitization is the absence of validated or widely recognized methods for identifying and characterizing chemical respiratory sensitizers [8], as well as for differentiating between respiratory and skin sensitizers [12,22].

Animal models, such as the local lymph node assay (LLNA), have been employed to evaluate respiratory sensitizers. Although primarily designed for skin sensitization testing, this model has offered some insights into the respiratory sensitization potential, but despite the adaptation to a respiratory LLNA [23], the data obtained could not be used to gain a deep understanding of the mechanism leading to respiratory sensitization. Additionally, *in vivo* tests have been criticized for their limitations in accurately predicting human responses, as species differences play a significant role in data interpretation [24]. In response to ethical concerns and the stringent need for accurate tests, many countries from various regions around the globe have phased out or implemented restrictions on animal testing on cosmetics. The regulatory agencies, therefore, promote the development of alternative *in vitro* methods to animal testing to evaluate the impacts of chemicals on human health. The European Union led this global movement by adopting the Cosmetics Regulation in 2009 [25]. Alternative methods that are utilized to assess the safety of cosmetic ingredients and final products fall under the category of new approach methodologies (NAMs) [26,27], a comprehensive descriptor for any non-animal assay, methodology, approach, or combination thereof that can be employed to provide input on chemical hazard and risk assessment [28]. NAMs consist of *in chemico*, *in silico* and *in vitro* methods, which can be used as stand-alone methodologies or can be comprised of integrated approaches to testing and assessment (IATAs), defined approaches (DAs) for data interpretation, and performance-based evaluation of test methods [29].

While significant progress has been made in the identification of skin sensitizers, resulting in the development and publication of a completely described AOP [11] in which every key event (KE) is supported by one or more NAMs, the progress in the development of NAMs for the identification of respiratory sensitizers has been comparatively slower. However, recent proposals have introduced notable additions to the AOP for respiratory sensitization [30].

The lung is a complex organ characterized by cellular heterogenicity, and the type of cells, culture conditions and exposure methodology are key factors that need to be properly addressed when designing and developing physiologically relevant *in vitro* human lung models for the assessment of xenobiotics’ effects upon inhalation [31]. Respiratory sensitization is a complex immunological process in which dendritic cells, the most effective antigen-presenting cells, play a central role. Their activation, identified as a common key event (KE3) in both the established skin sensitization and proposed respiratory sensitization AOPs [12], is crucial. Incorporating dendritic cells, either in monocultures or cocultures with bronchial cells and in the alveolo-capillary barrier *in vitro* models, is essential for advancing the understanding of the immunological mechanisms underlying respiratory sensitization. Dendritic cells can be derived from human peripheral blood or THP-1, a human monocytic leukemia-derived immortalized cell line, using granulocyte-macrophage colony-stimulating factor GM-CSF and interleukin 4 (IL-4) [32,33]. Established cell lines are commonly used as surrogates for dendritic cells in sensitization studies [34], and several *in vitro* assays have been developed with the aim of correctly classifying respiratory sensitizers [9]. Undifferentiated THP-1 cells, in particular, have been utilized in monoculture for both the development [35,36] and validation of the human cell line activation test (h-CLAT) assay for the evaluation of skin sensitizers [37]. Additionally, THP-1 cells in monoculture have been used in studies evaluating respiratory sensitizers [38,39,40,41]. The cluster of differentiation (CD)34^+^ human acute myeloid leukemia cell line (MUTZ-3) used undifferentiated in monoculture has also served as a dendritic cell model for the assessment of respiratory sensitizers [42]. In coculture systems, naïve THP-1 cells have been incorporated to study respiratory sensitizers [43,44,45].

Expression of CD86 and CD54 markers is measured on the surface of dendritic cells (DCs) to assess the skin sensitizing potential of chemicals in the h-CLAT assay [37]. Since skin and respiratory sensitization share certain mechanisms [12], measuring the expression of CD86 and CD54 cell surface markers was found suitable for the detection of respiratory sensitizers due to their roles in immune response modulation. As skin and respiratory sensitizers differ primarily in the type of T cell response they induce, with skin sensitizers triggering a T helper 1 (Th1) response and respiratory sensitizers eliciting a Th2 response, defining specific markers for the correct identification of respiratory sensitizers and differentiation from skin sensitizers is essential. Thymic stromal lymphopoietin (TSLP), a cytokine produced primarily by epithelial cells at barrier surfaces and also by dendritic cells [46,47], has been studied in the context of asthma [48]. It has been identified as a key regulator of the Th2 immune response. Dendritic cells are also direct targets of TSLP, as its receptor is highly expressed in specific populations of myeloid dendritic cells. When naïve allogeneic T cells are cocultured with TSLP-conditioned dendritic cells, they acquire an inflammatory Th2-like phenotype characterized by the production of cytokines associated with respiratory sensitization [46].

ALIsens^®^, a complex three-dimensional (3D) *in vitro* coculture alveolar model based on alveolar type II cells secreting lung surfactant (A549) [49,50], endothelial cells (EA.hy926) [51], dendritic-like cells (undifferentiated THP-1) [52] and macrophage-like cells (differentiated THP-1) was developed by Chary and collaborators, 2019 [43]. The model has been shown to correctly identify respiratory sensitizers from the class of acid anhydrides based on a set of biomarkers: thymic stromal lymphopoietin receptor (TSLPr), CD86 and CD54 cell surface marker, soluble mediators release in cell culture medium (cytokines and chemokines) and gene expression [43]. An increased expression of CD86, CD54, TSLPr and of the ligand for OX40 (OX40L) on the surface of dendritic cells from exposed *in vitro* models has been shown to correctly identify respiratory sensitizers [41,43,50,53]. An enhanced expression of CD54 and TSLPr cell surface markers was detected 24 h post-exposure to respiratory sensitizers. TSLPr expression increased upon respiratory sensitizers exposure, but remained unchanged after exposure to irritants, highlighting its specificity in detecting respiratory sensitizers [43].

Since skin sensitizers were not tested in ALIsens^®^, the specificity of this model for identifying respiratory sensitizers remains uncertain, including its ability to differentiate between respiratory and skin sensitizers [9]. Additionally, the most relevant biomarkers from the panel proposed in the development stage of the model needed to be identified. The increased expression of CD86, CD54 and TSLPr markers in the context of an alveolo-capillary barrier enhances the model’s ability to detect respiratory sensitizers, offering a reliable method to differentiate between sensitizers and other chemical exposures [43].

The aim of this study was to evaluate the response of ALIsens^®^ by analyzing the expression of CD86, CD54 and TSLPr cell surface markers following exposure to known skin sensitizers and non-sensitizers. Additionally, this investigation sought to identify specific marker(s) and establish a preliminary threshold that could enable accurate classification of respiratory sensitizers.

## 2. Materials and Methods

All materials (cell lines, cell culture reagents and ware, chemicals, flow cytometry reagents), equipment and software are presented in Table S1 in the Supplementary Material, indicating the catalog number, supplier and location information.

### 2.1. Reagents and Chemicals

#### 2.1.1. Cell Culture Reagents and Chemicals

Dulbecco’s Modified Eagle’s Medium (DMEM), high glucose, with GlutaMAX^TM^ supplement, Roswell Park Memorial Institute 1640 (RPMI-1640), high glucose, with GlutaMAX^TM^ supplement, Iscove’s Modified Dulbecco’s Medium (IMDM), Phosphate buffered saline (PBS) and TrypLE^TM^ Express Enzyme (1×) were purchased from Gibco^TM^ (Erembodegem, Belgium). Accutase was purchased from Invitrogen^TM^ ThermoFischer Scientific (Carlsbad, NM, USA). Fetal bovine serum (FBS) was purchased from Sigma-Aldrich (Overijse, Belgium). 4-(2-Hydroxyethyl) piperazine-1-ethanesulfonic acid (HEPES) (Chemical Abstracts Service Number (CAS No) 7365-45-9), phorbol-12-myristate-13-acetate (PMA) (CAS No 16561-29-8), resazurin sodium salt (CAS No 62758-13-8) were purchased from Sigma-Aldrich (Overijse, Belgium). 2-mercaptoethanol (β-ME) (CAS No 60-24-2) was purchased from Bio-Rad (Temse, Belgium).

#### 2.1.2. Chemicals Tested in the Study

Brij^®^ 35 surfactant was purchased from Merck Life Sciences, Hoeilaart, Belgium. Citric acid and lactic acid were purchased from VWR Chemicals, Leuven, Belgium. Dimethyl sulfoxide (DMSO) (CAS No 67-68-5), α-terpineol, 1,2-benzisothiazol-3(2H)-one, 2-mercaptobenzothiazole, benzylideneacetone, *trans*-cinnamaldehyde, chloroxylenol, diphenylcyclopropenone, ethylene glycol dimethacrylate, eugenol, imidazolidinyl urea, D-limonene, *p*-phenylenediamine were purchased from Sigma-Aldrich (Overijse, Belgium). The CAS No for the tested chemicals is provided in Table 1.

#### 2.1.3. Selection of Chemicals

The selection criteria for the tested chemicals were based on the availability of LLNA reference data, their use as proficiency chemicals in the Organisation for Economic Co-operation and Development Test Guidelines (OECD TGs) for skin sensitization, and the availability of results from *in chemico* and *in vitro* methods for skin sensitization, specifically the direct peptide reactivity assay (DPRA) [57] and the h-CLAT [37], respectively. Among the 11 selected skin sensitizer chemicals, 4 are classified as weak sensitizers, 4 are classified as moderate sensitizers, 1 as strong sensitizer and 1 as extreme sensitizer, according to LLNA (Table 1).

### 2.2. Cell Culture

The human cell lines A549, EA.hy926 and THP-1 were obtained from the American Type Culture Collection (ATCC^®^, Manassas, VA, USA). A549, EA.hy926 and THP-1 cells were cultivated as previously described [43]. Adherent cell lines, A549 and EA.hy926, were routinely subcultured twice a week using TrypLE^TM^ Express Enzyme (1×) and were used up to passage number 25. A549 cells were cultivated in DMEM supplemented with 10% FBS. EA.hy926 cells were cultivated in DMEM, supplemented with 10% FBS and 25 mM HEPES. The suspension cell line, THP-1, was routinely subcultured twice a week and was used up to passage number 25. THP-1 cells were cultivated in RPMI-1640 supplemented with 10% FBS and 50 µM β-ME. All cells were cultivated in a humidified incubator at 37 °C, with 5% CO_2_ and tested every 6 months for mycoplasma contamination using the MycoAlert^®^ Mycoplasma Detection Kit (Lonza, Roermond, The Netherlands). The identity of the cell lines was confirmed via human short tandem repeat (STR) profiling cell authentication service provided by ATCC^®^. A volume of approximately 40 µL cell suspension, containing at least 1 × 10^6^ cells, was spotted on the Flinders Technology Associates (FTA) sample collection card. The FTA cards were allowed to dry and then returned to ATCC^®^ to genotype the cell lines. The identity of the cells was confirmed by ATCC^®^.

### 2.3. ALIsens^®^ Test System Build-Up

**Figure 1:**
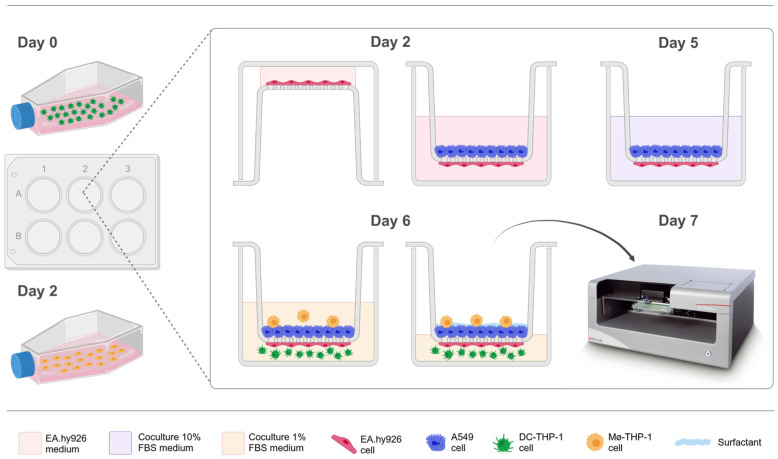
Schematic representation of the workflow for the build-up of ALIsens^®^ for the detection of respiratory sensitizers (adapted and modified from [43]) [61].

The 3D alveolar *in vitro* model, ALIsens^®^, was set up following the methodology proposed by Klein et al., 2013 [60], with the modifications implemented by Chary et al., 2019 [43] for the *in vitro* test system designed for the identification of respiratory sensitizers. The workflow schema is presented in Figure 1.

On day 0, THP-1 cells were seeded in T175 flasks (ThermoFischer Scientific, Merelbeke, Belgium) at a density of 4 × 10^5^ cells/mL in a complete THP-1 cell culture medium. Cells were induced to differentiate into macrophage-like THP-1 cells (MΦ-THP-1) by the addition of PMA at a concentration of 20 ng/mL. PMA was prepared as a stock solution (10 mg/mL) in ultrapure absolute ethanol and stored at −20 °C. This stimulation process was carried out for 48 h in a humidified incubator at 37 °C and 5% CO_2_. Subsequently, the PMA-supplemented medium was replaced with fresh THP-1 complete cell culture medium on day 2.

On day 2, 6-well cell culture plates (Greiner Bio-One, Vilvoorde, Belgium) were filled with hanging cell culture inserts, provided with a high pore density membrane with a surface area of 4.5 cm^2^ and 5 μm pore size (cellQART, Sabeu, Northeim, Germany). The plates with inserts were inverted to seed human endothelial cells, EA.hy926 (2.4 × 10^4^ cells/cm^2^), on the basolateral side of the insert semipermeable membrane. After the cells attached to the membrane, the plate with inserts was returned to its original orientation, and A549 epithelial cells were seeded on the apical side of the cell culture inserts (6 × 10^4^ cells/cm^2^). Complete EA.hy926 cell culture medium was added to the basolateral compartment, and both epithelial and endothelial cells were cultured to reach confluency over three days at 37 °C and 5% CO_2_.

On day 5, the cell culture medium was removed from both compartments and coculture medium consisting of a mix of 25 mM HEPES buffered DMEM (75%), RPMI-1640 (15%) and IMDM (10%) supplemented with 10% FBS (coculture 10% FBS medium) was added.

On day 6, undifferentiated dendritic-like THP-1 (DC-THP-1) cells (1 × 10^6^ cells/well) were seeded in the wells of new 6-well suspension cell culture plates (Greiner Bio-One, Vilvoorde, Belgium) using coculture medium consisting of a mix of HEPES buffered DMEM (75%), RPMI (15%) and IMDM (10%) supplemented with 1% FBS (coculture 1% FBS medium). The inserts containing the A549/EA.hy926 cells biculture were transferred to the plates containing the DC-THP-1 cells. MΦ-THP-1 cells were washed with PBS and were detached using Accutase for harvesting and seeding in the apical compartment (2.4 × 10^4^ cells/cm^2^) on the surface of the A549 cell monolayer. The medium for the tetraculture *in vitro* model was supplemented with 1% FBS to prevent excessive proliferation of DC-THP-1 cells. The attachment of MΦ-THP-1 cells to the epithelial monolayer was confirmed via light microscopy 4 h after seeding, and the medium was aspirated from the apical compartment. The test system was kept for 24 h at the air-liquid interface (ALI) before exposure.

### 2.4. Exposure

Exposure of ALIsens^®^ was carried out using the TECAN D300e Digital Dispenser and T8+ dispensehead cassettes (Tecan, Mechelen, Belgium). The test items were dispensed onto the apical surface of the test system using the D300e Pattern software (version 1). The dispenser accurately and reproducibly delivers solutions of test items solubilized in pure DMSO or sterile water containing 0.1% Brij^®^ 35 surfactant on the apical surface of the test system. The circular pattern was composed of 200 droplets of a volume of 30 nL each, arranged in a 0.5 × 0.5 mm grid format and spread over the apical surface area, with a total single dispense volume of 6 μL per insert. Prior to exposure to the selected test items, the impact of the two vehicles used for solution preparation on the viability of the test system was assessed. Pure DMSO and sterile water containing 0.1% Brij^®^ 35 surfactant were dispensed on the apical surface, and cell viability was assessed after 24 h of incubation concurrently with non-exposed inserts (incubator control). ALIsens^®^ was then exposed to increasing doses of the evaluated chemicals for 24 h to experimentally determine the individual dose-response curves for viability of the: (1) cells comprised in the apical compartment (A549+MΦ-THP-1), (2) EA.hy926 cells and (3) DC-THP-1 cells from the basolateral compartment and mathematically calculate the dose-response curves for cell viability of the (4) ALIsens^®^ model as an average of the first three individual dose-response curves. The dose-response curve of the ALIsens^®^ model was used to calculate the doses of investigated chemicals that reduce the cell viability of the test system by 25%, which were subsequently used to expose ALIsens^®^ for the evaluation of endpoints for respiratory sensitization. The minimum and maximum doses dispensed for each test item are presented in Table 1.

### 2.5. Endpoint Measurements

#### 2.5.1. Cell Viability

Cell viability of ALIsens^®^ was measured using the resazurin assay. The test system was exposed for 24 h to increasing doses (μg/cm^2^) of test items and the corresponding vehicle controls. Coculture 1% FBS medium was supplemented with 20 mM resazurin sodium salt stock solution prepared in PBS to reach a working concentration of 400 μM resazurin (resazurin working solution). The hanging cell culture inserts were transferred from the initial plates to new 6-well plates containing 1 mL of resazurin working solution to evaluate the viability of EA.hy926 cells from the tetraculture, and 1 mL of resazurin working solution was added in the apical compartment of the test system to evaluate the viability of A549 and MΦ-THP-1 cells from the tetraculture. To evaluate the viability of DC-THP-1 cells, 20 µL of resazurin stock solution was added to the cell suspension remaining in the wells of the plate and homogenized by horizontal manual shaking to reach a final concentration of 400 μM resazurin. The plates containing the test system exposed to test items, and the corresponding vehicle controls were incubated at 37 °C, 5% CO_2_, in the dark for 1 h. Following the incubation period, aliquots of coculture 1% FBS medium ± metabolized resazurin, resorufin, were sampled from the apical (A549+ MΦ-THP-1) and basolateral (EA.hy926) side of the hanging cell culture insert and from the well of the plate containing the DC-THP-1 cell suspension, and pipetted to a 96-well, flat-bottom microplate (Greiner Bio-One, Vilvoorde, Belgium). The absolute fluorescence intensity of resorufin was measured using the TECAN Spark 20M multi-mode microplate reader (Tecan, Mechelen, Belgium) at 530 nm excitation and 590 nm emission. Relative cell viability was calculated using Equation (1):

(1) $$\mathrm{Cell}\;\mathrm{viability}=\frac{\mathrm{Absolute}\;\mathrm{fluorescence}\;\mathrm{intensity}\;\mathrm{of}\;\mathrm{the}\;\mathrm{sample}-\mathrm{Blank}}{\mathrm{Absolute}\;\mathrm{fluorescence}\;\mathrm{intensity}\;\mathrm{of}\;\mathrm{the}\;\mathrm{vehicle}\;\mathrm{control}-\mathrm{Blank}}\times 100$$

The exposure dose reducing the viability of ALIsens^®^ by 25%, resulting in a 75% residual cell viability (CV75) was calculated using Equation (2):

(2) $$\mathrm{LogCV}75=\frac{\left(75-c\right)\times \;\mathrm{Log}\left(b\right)-\left(75-a\right)\times \;\mathrm{Log}\left(d\right)}{a-c}$$

where a is the minimum value of cell viability over 75%, c is the maximum percentage of cell viability below 75%, and b and d are the concentrations showing the percentage of cell viability at a and c, respectively [37].

#### 2.5.2. Flow Cytometry

DC-THP-1 cell suspensions were collected from the wells of the plates in conical centrifuge tubes (Greiner Bio-One, Vilvoorde, Belgium) and were centrifuged for 5 min at 300× *g*. The cell pellets were resuspended and washed in PBS at room temperature. The cells were centrifuged for 5 min at 300× *g*, the PBS wash was discarded, and the cells were resuspended in PBS supplemented with 2% FBS. Cells were stained at 4 °C for 45 min using commercially available fluorescent-labeled monoclonal antibodies: allophycocyanin (APC) mouse anti-human TSLPr (clone 1F11/TSLPR), Brilliant^TM^ Blue 515 (BB515) mouse anti-human CD54 (clone HA58) and phycoerythrin (PE) mouse anti-human CD86 (clone 2331 (FUN-1)), and κ isotype-matched controls (mouse IgG1), APC (clone MOPC-21), BB515 (clone X40) and PE (clone MOPC-21) (BD Biosciences, Erembodegen, Belgium). 1 μM SYTOX^TM^ Blue Nucleic Acid Stain (Invitrogen^TM^ ThermoFischer Scientific, Carlsbad, NM, USA) was used as a marker for non-viable cells. Expression of the cell surface markers was measured using the BD FACSCelesta^TM^ Cell Analyzer (BD Biosciences, Erembodegen, Belgium), and data was collected using the BD FACSDiva^TM^ software (version 9) provided with the instrument for data acquisition. A total of 10,000 events were acquired per sample, and further analysis was performed using FlowJo software (version 10) (BD Life Sciences, Ashland, OR, USA). Relative geometric mean fluorescence intensities (rMFI) were expressed as % compared to the vehicle control and calculated using Equation (3):

(3) $$\mathrm{rMFI}=\frac{\mathrm{MFI}\;\mathrm{sample}-\mathrm{MFI}\;\mathrm{isotype}\;\mathrm{control}\;\mathrm{of}\;\mathrm{the}\;\mathrm{sample}}{\mathrm{MFI}\;\mathrm{vehicle}\;\mathrm{control}-\mathrm{MFI}\;\mathrm{isotype}\;\mathrm{control}\;\mathrm{of}\;\mathrm{the}\;\mathrm{vehicle}\;\mathrm{control}}\times 100$$

### 2.6. Graphics and Language

**Figure 2:**
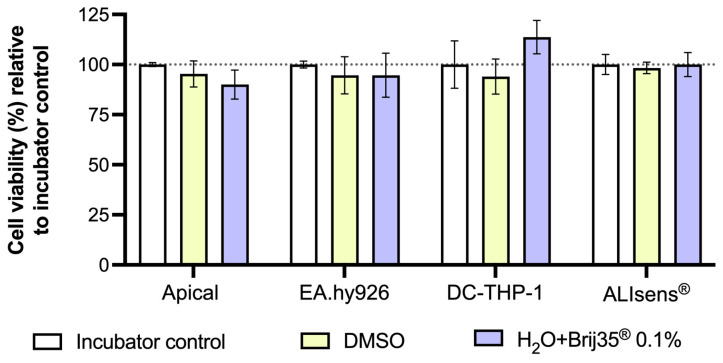
Viability of cells from the apical compartment (A549 and MΦ-THP-1), EA.hy926 and DC-THP-1 cells and ALIsens^®^ following exposure to pure DMSO and sterile water containing 0.1% Brij^®^ 35, relative to the incubator control. Data are expressed as mean ± SD of n = 1, N = 3. *p* values (>0.05) were determined by one-way ANOVA, Dunnett’s post-hoc.

**Figure 3:**
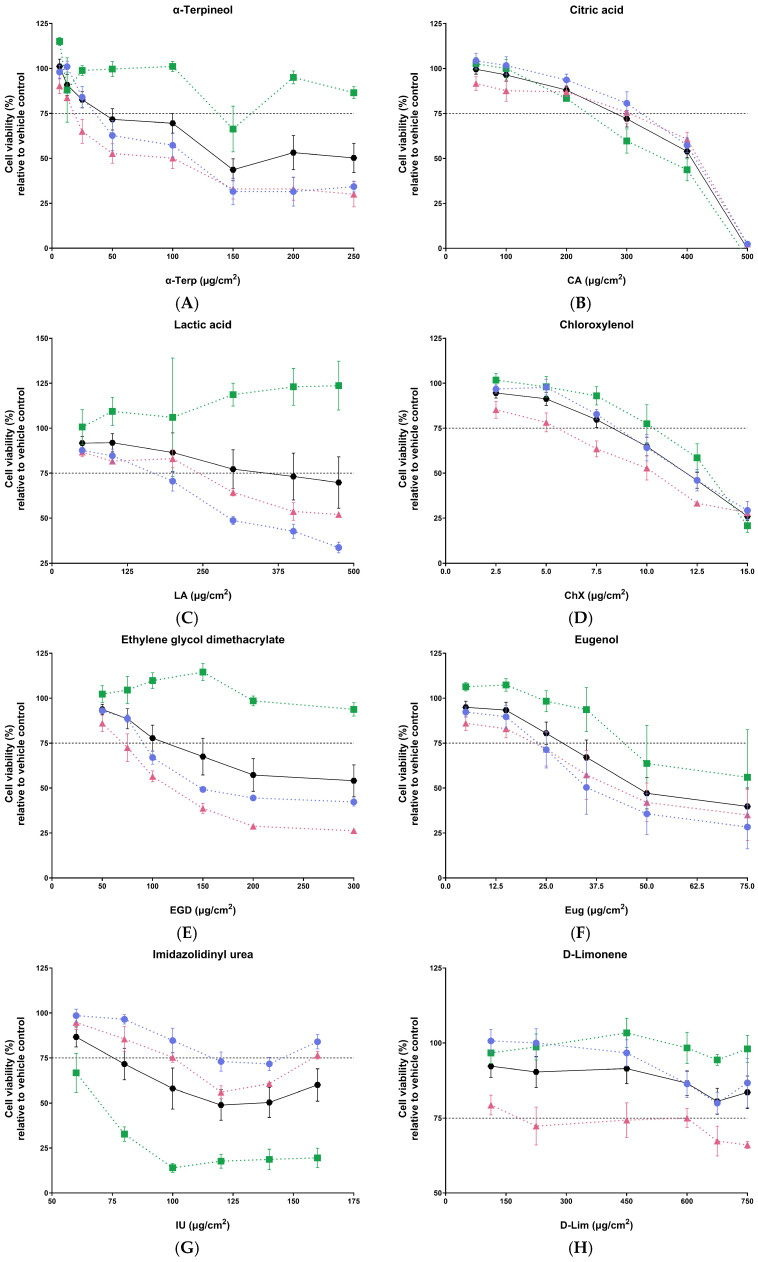

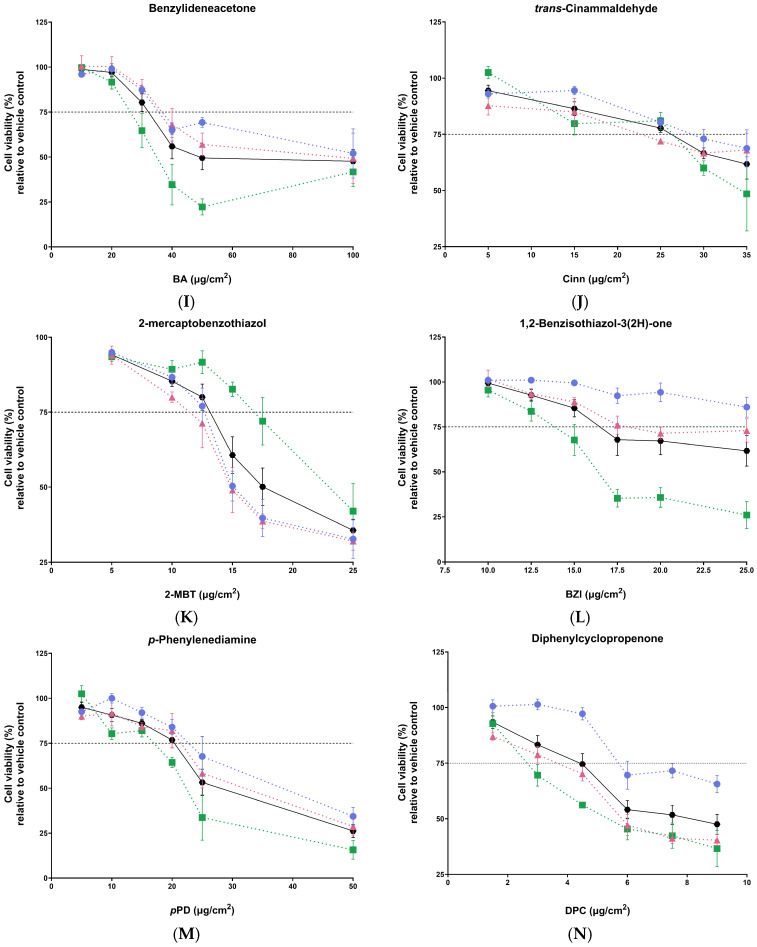
Viability of ALIsens^®^ (black hexagons and line), A549 and MΦ-THP-1 cells (blue circles and dotted line), EA.hy926 cells (pink triangles and dotted line) and DC-THP-1 cells (green squares and dotted line) relative to the corresponding vehicle control following 24 h exposure to the investigated chemicals (**A**) α-terpineol, (**B**) citric acid, (**C**) lactic acid, (**D**) chloroxylenol, (**E**) ethylene glycol dimethacrylate, (**F**) eugenol, (**G**) imidazolidynil urea, (**H**) D-limonene, (**I**) benzylideneacetone, (**J**) *trans*-cinnamaldehyde, (**K**) 2-mercaptobenzothiazol, (**L**) 1,2-benzisothiazol-3(2H)-one, (**M**) *p*-phenylenediamine, (**N**) diphenylcyclopropenone. Data are expressed as mean ± SEM of n = 1, N ≥ 3.

**Figure 4:**
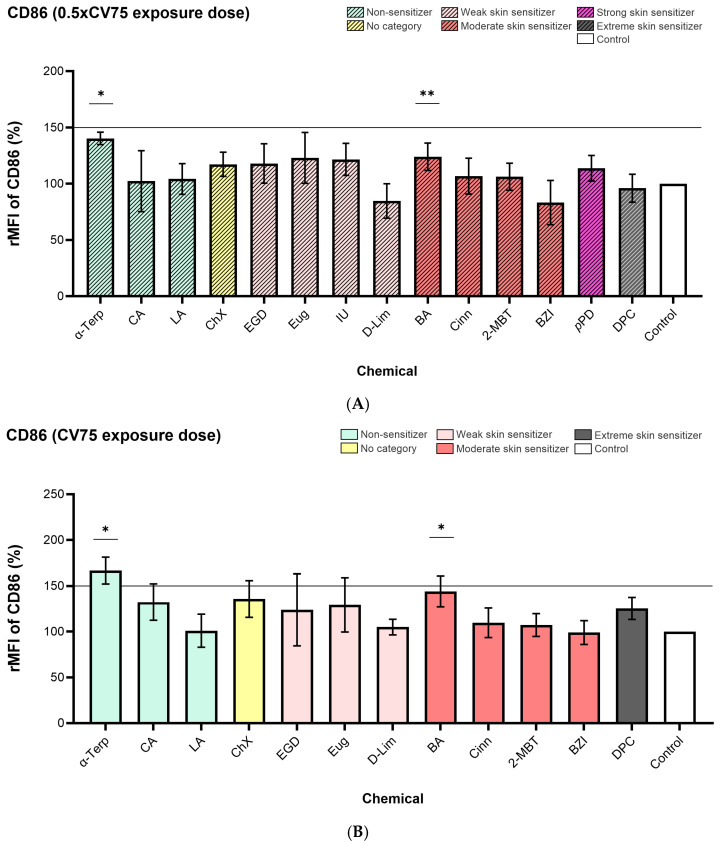
Expression of CD86 marker measured on the surface of DC-THP-1 cells following exposure of ALIsens^®^ for 24 h to (**A**) 0.5× CV75 dose and (**B**) CV75 dose of the evaluated chemicals, assessed by flow cytometry. Data are expressed as mean ± SD of n = 1, of N = 3 (α-Terp, CA, ChX, IU, D-Lim, DPC), N = 4 (LA, EGD, Eug, Cinn, 2-MBT, *p*PD), N = 5 (BZI); N = 6 (BA) for the 0.5× CV75 exposure dose and of N = 3 (α-Terp, CA, ChX, D-Lim, BA, DPC), N = 4 (LA, EGD, Eug, Cinn, 2-MBT, BZI) for the CV75 exposure dose. *p* values were determined by one-sample *t*-test. *p* ≤ 0.05 = *, *p* ≤ 0.01 = **.

**Figure 5:**
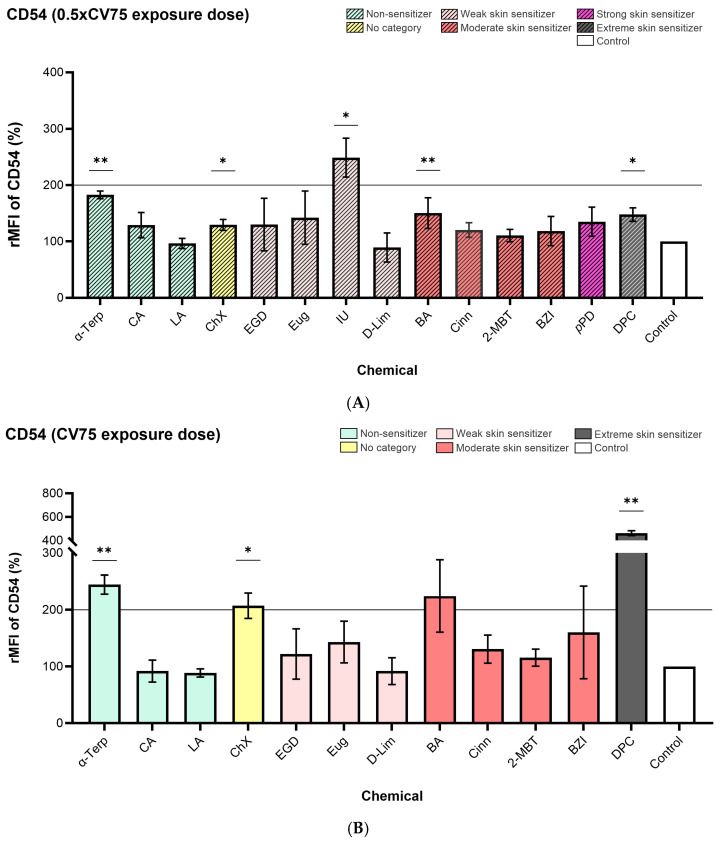
Expression of CD54 marker measured on the surface of DC-THP-1 cells following exposure of ALIsens^®^ for 24 h to (**A**) 0.5× CV75 and (**B**) CV75 of the evaluated chemicals, assessed by flow cytometry. Data are expressed as mean ± SD of n = 1, of N = 3 (α-Terp, CA, ChX, IU, D-Lim, DPC), N = 4 (LA, EGD, Eug, Cinn, 2-MBT, *p*PD), N = 5 (B ZI); N = 6 (BA) for the 0.5× CV75 exposure dose and of N = 3 (α-Terp, CA, ChX, D-Lim, BA, DPC), N = 4 (LA, EGD, Eug, Cinn, 2-MBT, BZI) for the CV75 exposure dose. *p* values were determined by one-sample *t*-test. *p* ≤ 0.05 = *, *p* ≤ 0.01 = **.

**Figure 6:**
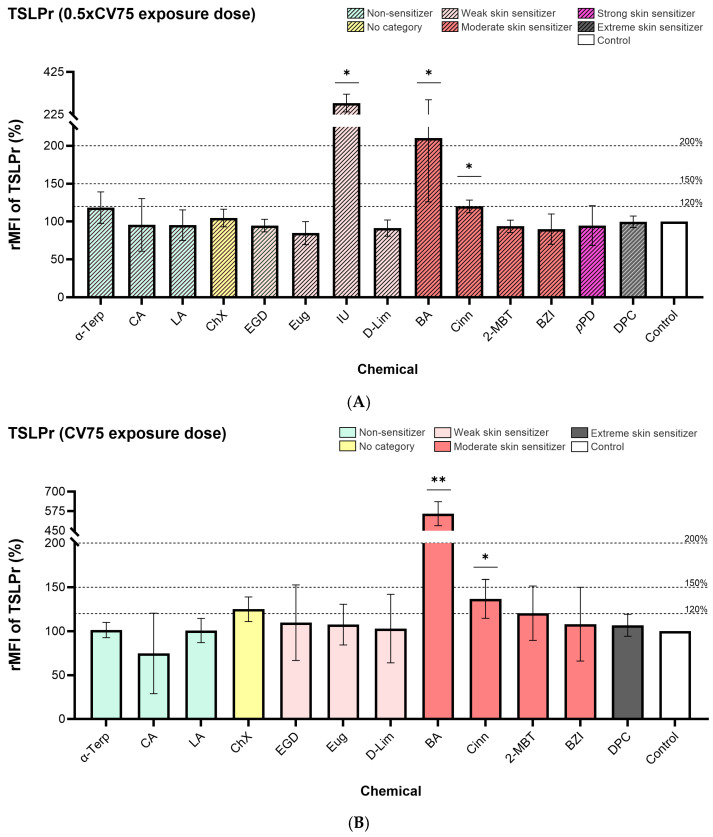
Expression of TSLPr marker measured on the surface of DC-THP-1 cells following exposure of ALIsens^®^ for 24 h to (**A**) 0.5× CV75 and (**B**) CV75 of the evaluated chemicals, assessed by flow cytometry. Data are expressed as mean ± SD of n = 1, of N = 3 (α-Terp, CA, ChX, IU, D-Lim, DPC), N = 4 (LA, EGD, Eug, Cinn, 2-MBT, *p*PD), N = 5 (BZI); N = 6 (BA) for the 0.5× CV75 exposure dose and of N = 3 (α-Terp, CA, ChX, D-Lim, BA, BZI, DPC), N = 4 (LA, EGD, Eug, Cinn, 2-MBT) for the CV75 exposure dose. *p* values were determined by one-sample *t*-test. *p* ≤ 0.05 = *, *p* ≤ 0.01 = **.

BioRender platform (Science Suite Inc., Toronto, ON, Canada) was used for the preparation of Figure 1. GraphPad Prism software (version 10) (GraphPad Software Inc., San Diego, CA, USA) was used for the preparation of Figure 2, Figure 3, Figure 4, Figure 5 and Figure 6. ChatGPT-3.5 artificial intelligence (AI) language model (OpenAI, San Francisco, CA, USA) was used to improve the language and readability of the manuscript. Mendeley Reference Manager software (version 2) (Elsevier, Amsterdam, The Netherlands) was used to prepare the list of references.

### 2.7. Statistical Analysis

Data are presented as mean ± standard deviation (SD) or standard error of the mean (SEM). The number of technical replicates (n), biological replicates (N), and statistical tests are indicated in the figures’ descriptions. Data from the cell viability dose-response curves was used to calculate the CV75 value. Differences between control and exposed groups were evaluated for the vehicle impact on the viability of ALIsens^®^ and cell surface marker expression and were considered statistically significant if *p*-values were *p* ≤ 0.05 = *, *p* ≤ 0.01 = **. Data were analyzed using GraphPad Prism software (version 10).

## 3. Results

### 3.1. Cell Viability

#### 3.1.1. Vehicle Impact on the Viability of ALIsens^®^

The test items solutions were prepared in pure DMSO and sterile water with 0.1% Brij^®^ 35 surfactant and were delivered on the apical surface of ALIsens^®^ using the TECAN D300e digital dispenser. DMSO is a vehicle frequently used for the dissolution of chemicals that are tested in cell culture and is usually used at a concentration that does not influence the test and the viability of the cell lines used in the method [62], being usually diluted in the exposure vehicle. Brij^®^ 35, a nonionic surfactant, was added to the aqueous solution to be dispensed to reduce the surface tension. Surfactants are irritants [63], and they can lead to cytotoxic effects in *in vitro* studies employing cell lines [64]. In our study, the solutions of tested chemicals were directly applied to the apical compartment of ALIsens^®^ in a pattern of 200 droplets, each containing 30 nL, covering the entire surface of the apical compartment. The impact of the vehicles used for the preparation of solutions of tested chemicals on the viability of the *in vitro* test system was evaluated against a non-exposed sample, the incubator control. Data reveals that direct application of pure DMSO and sterile water containing 0.1% Brij^®^ 35 surfactant did not have a significant impact on the viability of any of the cell types comprised in the *in vitro* alveolar test system (Figure 2).

#### 3.1.2. Dose-Response Curves of Evaluated Chemicals

The viability of ALIsens^®^ was evaluated 24 h post-exposure using the resazurin assay. This allowed the determination of the dose-response curve of each tested chemical that provided the data to calculate the final exposure doses (μg/cm^2^) for the analysis of sensitization potential. The standard ALIsens^®^ protocol requires that the respiratory sensitization potential is tested 24 h post-exposure using an exposure dose of the test item, leading to a 25% reduction of cell viability (residual cell viability of 75%), CV75 [43].

The cell viability assay has shown that all tested chemicals (Figure 3), with the exception of D-limonene (Figure 3H), reduced the viability of cells with increasing exposure dose. This allowed the calculation of the CV75 dose for the chemicals which reduced the viability of the test system by more than 25%. The calculated doses that were used for exposures for the evaluation of respiratory sensitization markers are presented in Table 2. As D-limonene did not reduce the cell viability of ALIsens^®^ under 75%, the highest exposure dose from the range of doses applied for the generation of the dose-response curve (750 µg/cm^2^) and 0.5× 750 µg/cm^2^ (375 µg/cm^2^) were used for the exposures for the assessment of respiratory sensitization endpoints.

Following exposure to increasing doses of citric acid (Figure 3B), chloroxylenol (Figure 3D), eugenol (Figure 3F), *trans*-cinnamaldehyde (Figure 3J), 2-mercaptobenzothiazol (Figure 3K), 1,2-benzisothiazol-3(2H)-one (Figure 3L), *p*-phenylenediamine (Figure 3M) and diphenylcyclopropenone (Figure 3N), all cell types comprised in the ALIsens^®^ model exhibit a uniform response, with the viability of each cell type steadily decreasing as the exposure dose increases. α-Terpineol (Figure 3A), chloroxylenol (Figure 3D), ethylene glycol dimethacrylate (Figure 3E) and D-limonene (Figure 3H) exposures had the highest impact on the EA.hy926 cells. The viability of DC-THP-1 cells does not consistently decrease with increasing doses of α-terpineol (Figure 3A), lactic acid (Figure 3C), ethylene glycol dimethacrylate (Figure 3E) and D-limonene (3H). Overall, these chemicals have minimal to no impact on the viability of DC-THP-1 cells. On the other hand, DC-THP-1 cells seem to be the most sensitive cells of the ALIsens^®^ model upon exposure to imidazolidinyl urea (Figure 3G), benzylideneacetone (Figure 3I), 1,2-benzisothiazol-2(3H)-one (Figure 3L) and diphenylcyclopropenone (Figure 3N).

### 3.2. Cell Surface Markers Expression

The CD86, CD54, and TSLPr cell surface markers expression measured on DC-THP-1 cells from the exposed ALIsens^®^ model are presented in Figure 4, Figure 5, and Figure 6, respectively.

α-Terpineol enhanced the expression of CD54 and CD86 markers on the surface of DC-THP-1 cells following exposure of the ALIsens^®^ model. Expression of CD86 (140% for 0.5× CV75 exposure dose and 167% for CV75 exposure dose) (Figure 4) and CD54 (183% for 0.5× CV75 exposure dose and 245% for CV75 exposure dose) (Figure 5) markers was significantly induced at both tested doses of α-terpineol, exceeding the 150% and 200% thresholds, respectively, at the CV75 exposure dose (Figure 4B and Figure 5B). No significant induction of the TSLPr cell surface marker was observed following exposure to the 0.5× CV75 and CV75 doses of α-terpineol, which resulted in an upregulation of 118% and 101% as compared to control, respectively (Figure 6A,B). Chloroxylenol significantly increased the expression of CD54 cell surface marker following exposure to both exposure doses (129% for 0.5× CV75 and 207% for CV75) (Figure 5A,B) and over the 200% threshold following exposure to CV75 exposure dose (Figure 5B). No significant increase in the expression of the other investigated markers, CD86 and TSLPr, was observed following exposure to the two doses of chloroxylenol (Figure 4 and Figure 6).

Imidazolidinyl urea exposure significantly increased the expression of CD54 (249%) and TSLPr (279%) markers and over the 200% threshold for the CD54 marker at 0.5× CV75 dose. Benzylideneacetone significantly enhanced the expression of CD54 cell surface marker at the lower exposure dose, 0.5× CV75 (150%) (Figure 5A), with an increase in expression over the 200% threshold following exposure to CV75 dose (Figure 5B). Both evaluated exposure doses of benzylideneacetone (0.5× CV75 and CV75) significantly induced the expression of CD86 (Figure 4A,B) and TSLPr (Figure 6A,B) cell surface markers.

*Trans*-Cinnamaldehyde significantly upregulated TSLPr expression on the surface of DC-THP-1 cells following exposure to both doses of 0.5× CV and CV75, resulting in values of 120% and 137%, respectively, as compared to control (Figure 6A,B).

Diphenylcyclopropenone significantly induced the expression of CD54 cell surface marker up to 463%, over the 200% threshold, at the CV75 exposure dose (Figure 5B).

For two evaluated chemicals, imidazolidinyl urea and *p*-phenylenediamine, the expression of the cell surface markers following exposure to the CV75 doses was not calculated, as the cell viability of DC-THP-1 cells measured with SYTOX^TM^ Blue in the flow cytometric readings, was lower than 75%). A low cell viability of the cells can lead to potential diffuse labeling of cytoplasmic structures as a result of cell membrane destruction [35], and this could render false-positive or false-negative results. Imidazolidinyl urea reduced the cell viability of the DC-THP-1 cells from ALIsens^®^ below 75% already at the first testing dose from the dose-response curve, 60 μg/cm^2^. It also significantly increased the expression of the CD54 cell surface marker at the 0.5× CV75 exposure dose (37.8 μg/cm^2^), surpassing the 200% threshold set by the h-CLAT assay. Additionally, this skin sensitizer significantly induced the expression of the TSLPr marker on the surface of DC-THP-1 cells up to the value of 279%.

The accuracy of the three proposed thresholds for TSLPr cell surface marker expression to correctly identify skin sensitizers and non-sensitizers as non-respiratory sensitizers was determined using data from all 14 evaluated chemicals at the 0.5× CV75 dose. For the CV75 dose, accuracy was calculated based on data from 12 chemicals, as the data from exposures to imidazolidinyl urea and *p*-phenylenediamine were not included due to low DC-THP-1 cell viability, which prevented the evaluation of cell surface marker expression. At the 0.5× CV75 dose, an accuracy of 79% was observed for the 120 threshold, while 86% accuracy was achieved for both the 150% and 200% thresholds. At the CV75 dose, the accuracy was 67% for the 120 thresholds and 92% for the 150% and 200% thresholds (Table 3).

## 4. Discussion

AOP 40 outlines the mechanisms of skin sensitization, providing comprehensive insights into the skin sensitization pathway, making it the gold standard in the field [11]. This AOP serves as a starting point for understanding respiratory sensitization [30]. It informs AOP 39 [65] by offering a well-established framework for studying the molecular and cellular processes that lead to sensitization, including KEs such as hapten binding to skin proteins, cellular activation, and cytokine release. Since the immune mechanisms underlying skin and respiratory sensitization share similarities along the events of the two AOPs, with the main difference between skin and respiratory sensitization being the type of adaptive immune responses [12], the detailed guidance in AOP 40 helps researchers apply these principles to respiratory outcomes in AOP 39, supporting the development of non-animal testing strategies for respiratory sensitization. Gaining further knowledge of the mechanisms involved in the process of respiratory sensitization is critical for advancing the development of accurate assays [30].

The adverse outcomes (AOs) from exposure to different sensitizers vary, with respiratory sensitizers causing allergic reactions in the respiratory system, leading to conditions like asthma, rhinitis, or other respiratory tract issues [8], while skin sensitizers trigger allergic reactions in the skin, resulting in allergic contact dermatitis (ACD) or eczema. The severity of AOs is closely linked to their effect on an individual’s health and quality of life. Since the impact of these two factors varies, along with the type of treatment required, it is important to distinguish between the two classes of sensitizers. In addition, sensitization in one system can potentially exacerbate sensitization in the other, leading to more severe allergic reactions overall.

The exposure route is another aspect that needs to be considered in the context of sensitization, as skin sensitizers typically induce sensitization upon skin contact, while respiratory sensitizers primarily induce sensitization through inhalation. There is considerable evidence suggesting that respiratory sensitization can be acquired as a result of skin exposure [10,66,67,68]. Accurate identification of respiratory sensitizers is crucial for implementing effective preventive measures and minimizing exposure risks in occupational and environmental settings. Over-classifying chemicals as respiratory sensitizers can lead to unnecessary costs from additional workplace controls or regulatory bans, while under-classification compromises worker safety. Therefore, distinguishing respiratory sensitizers from skin sensitizers and non-sensitizers/respiratory irritants is essential for balanced protection and cost management [9].

The validated *in vitro* test systems for the evaluation of skin sensitizers cannot differentiate between skin and respiratory sensitizers [22], and moreover, some respiratory sensitizers which have been classified as sensitizers in the LLNA, yield false-negative results in some of the methods used for the evaluation of skin sensitizers, such as phthalic anhydride, a respiratory sensitizer [13,21], which tested negative in the h-CLAT assay [56]. The primary focus of ALIsens^®^ is to correctly identify the respiratory sensitization hazard of LMW chemicals. The test system proved to correctly identify acid anhydrides as respiratory sensitizers based on the selection of biomarkers, either expressed on the surface of DCs or secreted into the cell culture medium from the basolateral compartment of the model [43]. Nevertheless, further chemicals from the reference lists of respiratory sensitizers [21,69,70] need to be evaluated to define the capacity and the accuracy of the model to identify such allergens.

In the development of ALIsens^®^, the decision of positive or negative with respect to respiratory sensitization was based on statistically significant differences in the expression of evaluated cell surface markers (CD54, CD86, TSLPr) measured on at least three biological replicates. In the h-CLAT prediction model, each test item is evaluated in at least two independent runs to derive a single prediction. If the first two runs are not concordant for at least one of the two markers, a third run is needed, and the final prediction is based on the majority result of the three individual runs. The upregulation of CD54 or/and CD86 markers of at least or greater than 200% and 150%, respectively, confirms THP-1 cell activation, representing KE3 [37], a common KE for skin and respiratory sensitization [10,12].

This study aimed to assess the response of ALIsens^®^ by analyzing changes in the expression of cell surface markers when exposed to skin sensitizers and non-sensitizers to evaluate the specificity of the model to correctly identify respiratory sensitizers and differentiate between skin and respiratory sensitizers. In particular, we sought to identify the most indicative marker(s) and propose a preliminary threshold that could accurately identify skin sensitizers, non-sensitizers and non-respiratory sensitizers. To achieve this, we tested a panel of chemical substances for their potential to increase the expression of CD86, CD54 and TSLPr cell surface markers on DC-THP-1 cells. The study included skin sensitizers of varying potency (weak, moderate, strong, and extreme) and non-sensitizers, most of which are proficiency chemicals from the OECD TG for the evaluation of skin sensitizers (lactic acid, ethylene glycol dimethacrylate, eugenol, imidazolidinyl urea, D-limonene, benzylideneacetone, *trans*-cinnamaldehyde, 2-mercaptobenzothiazole, *para*-phenylenediamine) [37,57,58]. TSLPr has been previously identified as a promising and reliable marker for correctly detecting respiratory sensitizers and distinguishing them from non-sensitizers [43]. However, since no skin sensitizers were included in previous studies, it remained unclear whether ALIsens^®^ could correctly identify respiratory sensitizers and potentially differentiate them from skin sensitizers and non-sensitizers [9,43].

Among the tested skin sensitizers, α-terpineol led to a significant increase in the expression of CD86 and CD54 cell surface markers at both exposure doses applied to ALIsens^®^ for 24 h. For terpineol, a mixture of the four isomers, α-, β-, γ- and terpin-4-ol, with the α-terpineol as principal constituent, there is no concern for skin sensitization [71]. In the h-CLAT assay, α-terpineol was found positive, as it enhanced the expression of CD54 cell surface marker, but in the “2 out of 3” integrated approach, it was classified as a non-sensitizer, as it scored negative [59]. Our findings for α-terpineol are in accordance with the results from the h-CLAT assay. α-terpineol did not enhance the expression of the TSLPr cell surface marker, neither in a significant manner nor over the tentative thresholds, indicating that ALIsens^®^ can correctly identify α-terpineol as a non-respiratory sensitizer. Imidazolidinyl urea, tested at the 0.5× CV75 exposure dose, significantly increased the expression of CD54 and TSLPr cell surface markers, surpassing the established threshold of 200% in the h-CLAT assay and the proposed tentative criterion of 200%, respectively. This skin sensitizer yielded positive results in the h-CLAT assay for both evaluated cell surface markers, CD86 and CD54 [56]. Benzylideneacetone elevated the expression of TSLPr on the surface of DC-THP-1 cells at both tested exposure doses, surpassing the tentative threshold of 200% for TSLPr at both the 0.5× CV75 and CV75 doses. Chloroxylenol increased TSLPr expression beyond the proposed 120% threshold at the CV75 exposure dose, and *trans*-cinnamaldehyde increased the expression of the TSLPr cell surface marker at both exposure doses, over 120%, though it remained under 150% rMFI value. 2-mercaptobenzothiazole increased the expression of TSLPr over the 120% proposed threshold, but only at the low tested dose.

In the development of the h-CLAT assay, three arbitrary thresholds, relative fluorescence intensity (RFI) values of 120%, 150%, and 200%, were selected to evaluate the assay’s ability to correctly distinguish skin sensitizers from non-sensitizers. Two independent experiments were conducted at two laboratories, each testing four doses for each test item. The mean RFI values for CD86 and CD54 cell surface markers and cell viability were determined from these two independent experiments to assess accuracy across the selected thresholds [36]. A similar approach was applied in this study, and the obtained data facilitated the calculation of ALIsens^®^ accuracy in identifying skin sensitizers and non-sensitizers based on the proposed thresholds. The ALIsens^®^ model was exposed to two evaluated doses of each chemical, with at least three biological replicates per condition, to assess the rMFI values for TSLPr, CD54, and CD86 cell surface markers.

For the tentative criterion rMFI of 120%, the lowest accuracy was observed for both exposure conditions (0.5× CV75 and CV75) with values of 79% and 67%, respectively. For the 150% and 200% tentative criteria, an accuracy of 86% was observed for the 0.5× CV75 exposure dose and 92% for the CV75 exposure dose. It has been previously observed that activation of dendritic cells requires a certain level of toxicity [72,73], and at the same time, respiratory sensitization is a threshold-based phenomenon [66]. The accuracy for the thresholds of 150% and 200% was consistent across both exposure doses, with the 150% threshold emerging as the most suitable for effectively identifying skin sensitizers and non-sensitizers as non-respiratory sensitizers in the ALIsens^®^ model.

Cell viability is the gatekeeper assay in the evaluation of immunotoxic effects, and as a subtoxic dose was successfully used for the evaluation of skin sensitizers [74], a robust approach should be used. The expression for the evaluated cell surface markers was not evaluated for two tested chemicals at the CV75 exposure dose due to the low cell viability of the DC-THP-1 cells in the flow cytometric analysis. Therefore, an alternative approach should be developed for the testing of chemicals that significantly reduce the viability of THP-1 cells used as a model for dendritic cells in the test system but have minimal to no impact on the other cell types (i.e., A549, EA.hy926) used for the build-up of ALIsens^®^. Calculating the CV75 solely from the DC-THP-1 cell viability data acquired following exposure of ALIsens^®^ to such chemicals would represent a potential solution to ensure, on the one hand, that the dendritic cells were exposed to the test item indirectly, through the alveolo-capillary barrier, while on the other hand, the population of viable cells is more or less 75%, in order not to hamper the measurements of cell surface markers.

The cell viability information is important in evaluating the potential of substances to induce sensitization in the respiratory tract since ALIsens^®^ addresses a biological mechanism, the modulation of the expression of cell surface phenotypic markers associated with the maturation of DC-THP-1 cells. Despite its simplicity, the cell viability assay showed the complexity of the tetraculture model, as the different types of cells of ALIsens^®^ respond in a particular way following exposure to low molecular weight chemicals belonging to different chemical classes. Delivery of test items in the apical compartment did not necessarily reduce the cell viability of the combination of epithelial and macrophage-like THP-1 cells to a greater extent than reducing the viability of cells from the basolateral compartment, endothelial or dendritic-like THP-1 cells. These data suggest that the different cellular components of a complex alveolar *in vitro* test system may have certain implications in the mechanism of respiratory sensitization. The dissection of the model to evaluate the independent contribution of each cell type of the alveolo-capillary barrier and their individual and orchestrated impact on the dendritic cells could help connect some dots in the mechanism of respiratory sensitization.

In this study, we evaluated the third KE of the respiratory sensitization AOP, highlighting that ALIsens^®^ is a promising complex 3D NAM for the evaluation of respiratory sensitization hazard. The cellular components and the architecture of the model designed for the identification of respiratory sensitization could also contribute to the differentiation of respiratory sensitizers from skin sensitizers and non-sensitizers based on the second KE (KE2), the activation of danger signals through the release of inflammatory cytokines and chemokines [10,43]. Secreted markers GM-CSF, macrophage inflammatory protein-3-alpha (MIP-3α)/CCL20 and IL-10 have been identified as predictive markers for the correct classification of acid anhydrides in a linear discriminant analysis [43]. Several other *in vitro* NAMs developed for the evaluation of chemicals to induce respiratory sensitization also addressed the KE2 of the AOP and measured the expression or secretion of cytokines and chemokines, including IL-6, IL-8, IL-18, monocyte chemoattractant protein-1 (MCP-1)/chemokine ligand (CCL)2, growth regulated oncogene-a (GRO-α)/C-X-C motif (CX)CL1, and regulated on activation, normal T cell expressed and secreted (RANTES)/CCL5, IL-1a, tumor necrosis factor (TNF-α) [41,75,76,77]. As respiratory sensitization can be acquired following skin exposure, NAMs dedicated to the detection of skin sensitizers could be used to classify the two types of sensitizers and additionally provide a deeper understanding of the differences in the molecular mechanisms of respiratory and skin sensitization. Specifically, an *in vitro* study using keratinocytes as a cellular model evaluating IL-18 levels following exposure to skin and respiratory sensitizers concluded that the two evaluated respiratory sensitizers did not increase the intracellular cytokine concentration [78]. A panel of inflammatory markers, including, but not limited to, cytokines and chemokines previously identified as promising markers for the identification of respiratory sensitizers, could be incorporated in future studies to evaluate the response of ALIsens^®^ following exposure to respiratory and skin sensitizers as well as non-sensitizer.

## 5. Conclusions

The correct identification of respiratory sensitizers and differentiating them from skin sensitizers is crucial for the protection of human health, ensuring regulatory compliance, promoting occupational safety, and facilitating informed decision-making in various contexts, including product labeling and medical care.

By conducting this study, our aim was to answer several questions: (a) can the test system correctly identify skin sensitizers and non-sensitizers and potentially differentiate them from respiratory sensitizers? (b) what is/are the specific biomarker(s) that should be measured to correctly classify chemicals as respiratory sensitizers, and (c) what is the threshold for this/these biomarker(s) that provide the best specificity? Based on the obtained results and the calculated accuracy values for the TSLPr cell surface marker, we can conclude that ALIsens^®^ has the potential to identify skin sensitizers and not harmful chemicals as non-respiratory sensitizers. However, since no respiratory sensitizers were included in this study, it is essential to further validate the tentative 150% threshold with a larger dataset comprising data of respiratory sensitizers tested in ALIsens^®^ to ensure robust conclusions.

These findings can fill a knowledge gap in the AOP for respiratory sensitization and provide insights that can inform both the scientific community and the regulatory bodies. The findings of this research have the potential to pave the way towards a better understanding of the mechanisms of respiratory sensitization, ultimately contributing to the identification of respiratory sensitizers in the early development phases of novel chemicals and molecules across various industries, with the main goal of protecting human health.

## Figures and Tables

**Table 1:** Chemicals tested in the study.

Chemical	Acronym	CAS No ^a^	Molecular Weight (g/mol)	Vehicle for Stock Solution Preparation	Exposure Doses (µg/cm^2^)		Potency Category	h-CLAT ^e^		Proficiency/Reference Chemical OECD TG ^f^
					Minimum	Maximum		CD86	CD54	
α-Terpineol	α-Terp	98-55-5	154.25	DMSO	6.25	250	Non-sensitizer ^b^	- ^¥^	- ^¥^	n/a
Citric acid	CA	77-92-9	192.12	Water+ 0.1% Brij^®^ 35	50	500	No category	-	-	
Lactic acid	LA	50-21-5	90.08	DMSO	50	475	Non-sensitizer ^c^	-	-	442C, 442D, 442E
Chloroxylenol	ChX	88-04-0	156.61		2.5	15	No category	NA	NA	n/a
Ethylene glycol dimethacrylate	EGD	97-90-5	198.22		50	300	Weak ^c^	-	+	442D, 442E
Eugenol	Eug	97-53-0	164.20		5	75		+	+	
Imidazolidinyl urea	IU	39236-46-9	388.29		60	160		+	+	442E
D-Limonene	D-Lim	5989-27-5	136.23		112.5	750		-	+	
Benzylideneacetone	BA	122-57-6	146.19		10	100	Moderate ^c^	+	+	442C
*trans*-Cinnamaldehyde	Cinn	14371-10-9	132.16		5	35		n/a *	n/a *	442C, 442D
2-Mercaptobenzothiazole	2-MBT	149-30-4	167.25		5	25		-	+	442D, 442E
1,2-Benzisothiazol-3(2H)-one	BZI	2634-33-5	151.19		10	25		-	+	n/a
*p*-Phenylenediamine	*p*PD	106-50-3	108.14		5	50	Strong ^c^	+	-	442D, 442E
Diphenylcyclopropenone	DPC	886-38-4	206.24		1.5	9	Extreme ^d^	-	+	n/a

^a^ CAS No (Chemical Abstracts Service Number). ^b^ PLNA (Popliteal Lymph Node Assay) [54,55]. ^c^ LLNA (Local Lymph Node Assay) [56]. ^d^ Potency category classification [56]. ^e^ h-CLAT: +, sensitizer; -, non-sensitizer [56]. ^f^ OECD TG—Organisation for Economic Co-operation and Development Test Guideline: 442C [57], 442D [58], 442E [37]. ^¥^ CD86, CD54 data: [59]. * Cinnamic aldehyde (CAS 104-55-2): h-CLAT—CD54+ and CD86+ [56]. n/a—not applicable. NA—not available.

**Table 2:** Calculated 75% cell viability (CV75) exposure dose value and 0.5× CV75 exposure dose values for ALIsens^®^ exposed for 24 h to the evaluated chemicals.

Chemical	CV75 ^1^ (µg/cm^2^)	0.5× CV75 ^2^ (µg/cm^2^)
α-Terpineol	41.4	20.7
Citric acid	278	139
Lactic acid	346.4	173.2
Chloroxylenol	8.25	4.13
Ethylene glycol dimethacrylate	112.9	56.5
Eugenol	28.5	14.2
Imidazolidynil urea	75.5	37.8
D-Limonene	750 *	375 **
Benzylideneacetone	31.9	15.9
*Trans*-Cinnamaldehyde	26.2	13.1
2-Mercaptobenzothiazol	13.1	6.6
1,2-Benzisothiazol-3(2H)-one	16.4	8.2
*p*-Phenylenediamine	20.4	10.2
Diphenylcyclopropenone	4.62	2.31

^1^ The exposure doses resulting in 75% residual cell viability of the 24 h exposed ALIsens^®^, expressed in μg/cm^2^. ^2^ The exposure doses multiplied with a factor of 0.5 resulting in 75% residual cell viability of the 24 h exposed ALIsens^®^, expressed in μg/cm^2^. *—maximum exposure dose; **—0.5× maximum exposure dose.

**Table 3:** Summary data for TSLPr expression following exposure to the investigated chemicals to 0.5× CV75 and CV75 exposure doses.

Exposure Dose (µg/cm^2^)	0.5× CV75			CV75		
Tentative Threshold (%)	120	150	200	120	150	200
α-Terp	-	-	-	-	-	-
CA	-	-	-	-	-	-
ChX	-	-	-	+	-	-
LA	-	-	-	-	-	-
EGD	-	-	-	-	-	-
Eug	-	-	-	-	-	-
IU	+	+	+	n/a	n/a	n/a
D-Lim	-	-	-	-	-	-
BA	+	+	+	+	+	+
Cinn	+	-	-	+	-	-
2-MBT	-	-	-	+	-	-
BZI	-	-	-	-	-	-
*p*-PD	-	-	-	n/a	n/a	n/a
DPC	-	-	-	-	-	-
Accuracy (%)	79	86	86	67	92	92

rMFIs (%) of TSLPr expression on the surface of DC-THP-1 cells were compared to three tentative criteria: 120%, 150% and 200%. + represents a rMFI value of TSLPr ≥ tentative criterion, and “-” means a rMFI value of TSLPr < tentative criterion. n/a represents not available data.

## Data Availability

Data are available in the article.

## Supplementary Materials

The following supporting information can be downloaded at: https://www.mdpi.com/article/10.3390/toxics13010029/s1, Table S1. List of materials used in the study.
