# Supplementary material for: Responses of an In Vitro Coculture Alveolar Model for the Prediction of Respiratory Sensitizers (ALIsens®) Following Exposure to Skin Sensitizers and Non-Sensitizers
Source: Toxics. 2024 Dec 31;13(1):29. doi: 10.3390/toxics13010029 (PMC11769448; doi:10.3390/toxics13010029)
Supplement: Supplementary file 1 [file toxics-13-00029-s001.zip › toxics-3324960-supplementary.pdf]

Table S1. List of materials used in the study.

Material		Catalog number	Supplier	Location
Cell lines	A549	CCL-185	ATCC®	Manassas, VA, US
	EA.hy926	CRL-2922		
	THP-1	TIB-202		
	FTA Sample Collection Kit for Human Cell Authentication Service	135-XV-5		
	MycoAlert® Mycoplasma Detection Kit	LT07-318	Lonza	Roermond, Netherlands
Cell culture	Dulbecco's Modified Eagle's Medium (DMEM), high glucose, GlutaMAX™ supplement	61965026	Gibco™	Erembodegem, Belgium
	Roswell Park Memorial Institute 1640 (RPMI), high glucose, GlutaMAX™ supplement	61870036		
	Iscove's Modified Dulbecco's Medium (IMDM)	12440053		
	Phosphate buffered saline (PBS)	14190094		
	TrypLE™ Express Enzyme (1X), no phenol red	12604013		
	Fetal Bovine Serum (FBS) Superior	F0615	Sigma-Aldrich	Overijse, Belgium
	Trypan Blue (0.4%)	T8154		
	Accutase	00-4555-56	Invitrogen™ ThermoFischer Scientific	Carlsbad, NM, US
	C-Chip, Disposable Haemocytometer	DHC	Dulis	Namur, Belgium
Cell Culture Ware	Nunc™ EasYFlask™ Cell Culture Flasks 75 cm²	156499	ThermoFischer Scientific	Merelbeke, Belgium
	Nunc™ EasYFlask™ Cell Culture Flasks 175 cm²	159910		
	CellStar® Polystyrene 6-Well Cell Culture Plate	657160	Greiner Bio-One	Vilvoorde, Belgium
	CellStar® Polystyrene 6-Well Suspension Cell Culture Plate	657185		
	CellStar® Polystyrene 96-Well, Flat-Bottom, Cell Culture Microplate	655101		
	6-Well Insert, 5 µm PET clear	9305012	cellQART® made by SABEU	Northeim, Germany
	Falcon™ 96-Well, Non-Treated, V-Shaped-Bottom Microplate	734-0391	VWR	Leuven, Belgium
Chemicals	2-Mercaptoethanol	1610710	Bio-Rad	Temse, Belgium
	Brij® 35 solution	1.01894.0100	Merck Life Sciences	Hoeilaart, Belgium
	Citric acid	20282.293	VWR chemicals	Leuven, Belgium
	Lactic acid	101384Q		
	α-Terpineol	432628	Sigma-Aldrich	Overijse, Belgium
	1,2-Benzisothiazol-3(2H)-one	561487		
	2-Mercaptobenzothiazole	M3302		
	4-(2-Hydroxyethyl)piperazine-1-ethanesulfonic acid (HEPES)	H4034		

Chemicals	Benzylideneacetone	147885	Sigma-Aldrich	Overijse, Belgium
	trans-Cinnamaldehyde	C80687		
	Chloroxylenol	C38303		
	Dimethyl Sulfoxide (DMSO)	D2438		
	Diphenylcyclopropenone	177377		
	Ethylene glycol dimethacrylate	335681		
	Eugenol	E51791		
	Imidazolidinyl urea	I5133		
	D-Limonene	183164		
	p-Phenylenediamine	P6001		
	Phorbol 12-myristate 13-acetate (PMA)	79346		
	Resazurin sodium salt	R7017		
Flow cytometry	SYTOX™ Blue Nucleic Acid Stain	S34857	Invitrogen™ ThermoFischer Scientific	Carlsbad, NM, US
	BD Pharmingen™ APC Mouse Anti-Human TSLP Receptor (clone 1F11/TSLPR)	563153	BD Biosciences	Erembodegen, Belgium
	BD Horizon™ BB515 Mouse Anti-Human CD54 (clone HA58)	564685		
	BD Pharmingen™ PE Mouse Anti-Human CD86 (clone 2331 (FUN-1))	555658		
	BD Pharmingen™ APC Mouse IgG1, κ Isotype Control (clone MOPC-21)	555751		
	BD Horizon™ BB515 Mouse IgG1, κ Isotype Control (clone X40)	564416		
	BD Pharmingen™ PE Mouse IgG1, κ Isotype Control (clone MOPC-21)	555749		
Equipment	BD FACSCelesta™ Cell Analyzer (Flow cytometer)	n/a	BD Biosciences	Erembodegen, Belgium
	TECAN Spark 20 M (Fluorescence microplate reader)		TECAN	Mechelen, Belgium
	TECAN D300e (Digital dispenser)			
	T8+ dispensehead casette			
Software	BD FACS Diva™	n/a	BD Life Sciences	Ashland, OR, US
	FlowJo version 10			
	GraphPad Prism version 10		GraphPad Software Inc.	San Diego, CA, US
	Tecan Spark		Tecan	Mechelen, Belgium
	D300e Pattern			
	BioRender		Science Suite Inc.	Toronto, ON, Canada
	ChatGPT-3.5		OpenAI	San Francisco, CA, US
	Mendeley Reference Manager		Elsevier	Amsterdam, Netherlands

n/a – not applicable
